# Precision measurement of stratum corneum thickness in OCT images using variational autoencoders and advanced DSP techniques

**DOI:** 10.3389/fbioe.2025.1732519

**Published:** 2026-01-15

**Authors:** Haiyu Qin, Yang Wang

**Affiliations:** 1 Department of Electrical and Electronic Engineering, University of Sheffield, Sheffield, United Kingdom; 2 Department of Nursing, Faculty of Medicine, University Kebangsaan Malaysia, Kuala Lumpur, Malaysia

**Keywords:** dermatologic AI applications, digital signal processing, OCT skin imaging, stratum corneum thickness, variational autoencoders

## Abstract

Optical coherence tomography (OCT) has emerged as a cornerstone technique for *in vivo* skin imaging; however, reliable and clinically meaningful quantification of stratum corneum (SC) thickness remains challenging. This review summarizes 2 decades of methodological evolution, highlighting the transition from early manual and rule-based approaches to modern deep learning–driven segmentation strategies. Particular emphasis is placed on recent hybrid frameworks that integrate physics-informed digital signal processing with generative deep learning models, which collectively improve boundary detection robustness, reduce annotation dependency, and enhance model interpretability. These advances have significantly expanded the clinical utility of OCT-based SC assessment, enabling more sensitive disease monitoring, improved evaluation of therapeutic and cosmetic interventions, and broader applications in dermatologic diagnostics. Finally, we outline emerging opportunities for real-time, marker-free analysis, multimodal data fusion, and the development of explainable and generalizable algorithms to support precision and personalized dermatologic care.

## 1 Introduction

The stratum corneum (SC), the outermost layer of human skin, is a thin yet functionally critical structure that governs epidermal permeability, water retention, and protection against pathogens, allergens, and xenobiotics (Lintzeri et al., 2022). Although typically only 10–20 µm thick, the SC exerts a disproportionate influence on skin barrier function through its lipid–protein “brick-and-mortar” architecture. Even micrometre-scale deviations in SC thickness can substantially compromise barrier integrity or, conversely, modulate the penetration of therapeutic and cosmetic agents. Consequently, precise and reproducible quantification of SC thickness has emerged as a key objective in clinical dermatology, transdermal drug delivery, and personal-care science (You et al., 2023). However, accurately delineating this ultrathin, heterogeneous layer *in vivo* remains a major technical challenge, limiting the translation of advanced imaging modalities into routine clinical and industrial practice (Wang et al., 2024).

Optical coherence tomography (OCT) has become a leading non-invasive tool for SC assessment due to its depth-resolved imaging capability, micrometre-scale axial resolution (1–5 µm), and penetration depths approaching 1.8 mm in keratinized tissue (Lin et al., 2024). Modern spectral-domain, swept-source, and line-field confocal OCT (LC-OCT) systems can clearly distinguish the highly scattering SC from the underlying viable epidermis while preserving tissue integrity for longitudinal monitoring (Lintzeri et al., 2022). Unlike histology, OCT avoids fixation-induced shrinkage; unlike high-frequency ultrasound (Luan et al., 2023), it resolves fine epidermal layers without contrast agents; and unlike reflectance confocal microscopy (Yu et al., 2023b), it provides subsurface imaging over hundreds of micrometres at video rates. These advantages position OCT as an ideal modality for quantitative SC thickness mapping (Yang et al., 2024).

Despite these intrinsic strengths, the clinical and translational utility of OCT is constrained by a persistent bottleneck: the reliable extraction of SC boundary coordinates from raw OCT reflectivity data (Lin et al., 2024). Manual caliper-based measurements are subjective, labor-intensive, and poorly reproducible, with inter-observer variability often exceeding 15% (He et al., 2024). Histological validation, frequently treated as a reference standard, introduces tissue shrinkage artifacts of 12%–21%, undermining direct comparison with *in vivo* OCT measurements (Kerns et al., 2008). Automated segmentation approaches, including graph-search methods and convolutional neural network–based pipelines, have improved accuracy but remain limited by high computational demands, sensitivity to device-specific signal characteristics, and a heavy reliance on large, densely annotated training datasets (Kerns et al., 2008). These constraints hinder deployment in point-of-care settings, multicenter clinical trials, and large-scale cosmetic testing, where robustness, efficiency, and data economy are essential (Chen et al., 2025).

At the same time, the biological and clinical importance of precise SC thickness measurement continues to intensify (Gambichler et al., 2006b). Deviations from physiological SC thickness are closely associated with skin disorders (Xu et al., 2025) such as atopic dermatitis, xerosis, ichthyoses, and psoriasis, where barrier dysfunction, rather than thickness alone, correlates with disease severity and increased transepidermal water loss (Lin et al., 2024). Beyond pathology, controlled modulation of SC thickness through hydration, exfoliation, or formulation design is central to transdermal delivery and cosmetic efficacy; shifts of only 2–4 µm can alter active ingredient penetration by tens of percent. These effects are further complicated by pronounced inter-individual and anatomical variability, with SC thickness accounting for much of the epidermal thickness range observed across body sites, ages, phototypes, and environmental conditions. Detecting such subtle, early-stage changes requires measurement strategies that are both highly sensitive and biologically consistent.

To overcome these challenges, a shift is needed from purely discriminative, data-hungry segmentation pipelines toward models that explicitly integrate OCT physics with data-efficient representation learning (Tang et al., 2024). Physics-informed digital signal processing (DSP) techniques, such as speckle reduction, depth-dependent sensitivity compensation, dispersion correction, and deconvolution, can suppress imaging artifacts and linearize OCT signals before learning, reducing the burden placed on downstream models (Ozcan et al., 2007). Building on this foundation, variational autoencoders (VAEs) offer a powerful generative framework for SC analysis (Li et al., 2025). By learning continuous, regularized latent representations of skin morphology, VAEs can disentangle biologically meaningful factors, such as SC thickness and surface roughness, from noise and device-specific variability (Li K. et al., 2024). Their generative nature enforces structural coherence, reduces dependence on exhaustive pixel-level annotations, and enables intrinsic uncertainty estimation—features that are particularly valuable for clinical trust, cross-device generalization, and longitudinal monitoring.

This review synthesizes 2 decades of progress in SC thickness quantification with a particular focus on the emerging convergence of advanced DSP and VAE-based generative learning. We first outline the biological and clinical rationale for accurate SC thickness mapping and critically examine the limitations of conventional and contemporary measurement approaches. We then detail DSP strategies tailored to skin OCT and VAE architectures optimized for SC boundary delineation, benchmarking their performance against classical algorithms and discriminative deep learning models. Finally, we discuss current clinical and industrial applications, unresolved methodological challenges, and future directions, outlining a roadmap toward centimetre-scale, micrometre-accurate *in vivo* mapping of the human skin barrier. By integrating principles from optical physics, signal processing, and representation learning, this work aims to advance precision skin barrier assessment and support the development of personalized dermatological and cosmetic interventions.

## 2 Conventional methods for thickness estimation

**FIGURE 1:**
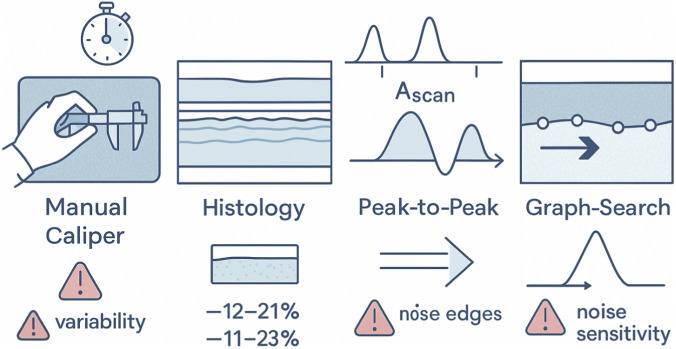
Schematic summary of conventional SC thickness-estimation methods.

The Early studies treated the SC reference standard as a straightforward length: place calipers on the OCT screen or excise a biopsy, embed it in paraffin, and read the distance between the corneocyte surface and the viable epidermis. Yet, each of these “conventional” routes introduces measurement error that is now well-documented (Gambichler et al., 2006a; Lintzeri et al., 2022). The manual placement of electronic calipers on individual B-scans remains the most widely used clinic-side technique because it requires no dedicated software. Unfortunately, it is both labor-intensive and operator-dependent. A recent multi-site trial that timed 1,587 manual measurements reported a mean reading time of 42 s per frame, an impractically burdensome task when hundreds of frames are generated in a 2-min sweep (Figure 1). The same study showed inter-observer coefficients of variation approaching 13% for forearm skin, even after a joint training session, largely because speckle noise masks the dermal-epidermal junction and encourages subjective placement of the basal line (Gambichler et al., 2006b). Manual protocols also require the user to down-sample three-dimensional stacks to a handful of “representative” slices, thereby forfeiting information on regional undulations and biasing group statistics, especially at acral or aged sites where papillary relief is pronounced (Jain et al., 2024).

Histology is often considered a gold standard, but its thickness numbers are themselves moving targets. From the moment a 4 mm punch is taken, contractile forces and subsequent processing steps initiate cumulative shrinkage. Optical tracking and serial caliper studies indicate that the loss in length is approximately 12%–21% and in width is 11%–23% before the slide even reaches the microscope (Kerns et al., 2008).​ Lipid extraction during dehydration collapses intercellular spaces within the SC, while paraffin infiltration hardens the sample, exaggerating the effect. Cryosectioning reduces shrinkage but sacrifices the crisp nuclear detail dermatopathologists prefer. The net result is that histological SC often reads several micrometres thinner than its *in-vivo* counterpart, forcing investigators to apply empirical correction factors that vary with fixation protocol, anatomic site, and patient age (Sandby-Møller et al., 2003; Kerns et al., 2008; Tran et al., 2015).

To circumvent these limitations, classical image-processing pipelines emerged in the mid-2000s. The simplest exploit the bimodal intensity profile of SD-OCT A-scans: taking the first strong reflection as the air/SC interface and a second peak as the dermal-epidermal junction. While fast, this “peak-to-peak” heuristic fails whenever the second maximum corresponds instead to a collagen bundle or papillary tip, resulting in systematic under-reads in more than 25% of scans (Gambichler et al., 2006a). Edge-based algorithms refine the approach by applying Sobel or Canny operators to each frame and then fitting polynomial splines through the strongest gradient. However, they struggle in low-contrast regions and tend to propagate single-frame errors through the spline, inflating the root-mean-square (RMS) error to 8–12 µm on healthy volar skin. The model-based methods improve robustness by incorporating anatomical priors. Active-contour “snakes” initialise near the skin surface and evolve toward energy minima that penalise curvature while rewarding high gradient magnitude. However, convergence stalls in the presence of speckle voids and requires careful tuning of elasticity parameters for each device. Graph-search formulations overcome many of these issues: they treat the B-scan as a weighted graph in which the cost of traversing a pixel is inversely proportional to its edge strength, then solve a shortest-path problem that yields globally optimal, smooth boundaries. Three-dimensional extensions that couple adjacent B-scans have reduced segmentation error by ∼20% relative to 2-D approaches and generate continuous thickness maps suitable for roughness assessment (Srivastava et al., 2018).

Despite these refinements, conventional image-processing pipelines share common weaknesses. First, most rely on handcrafted thresholds or filter kernels that were calibrated on homogeneous laboratory datasets, degrading when confronted with lower signal-to-noise ratios, darker phototypes, or atypical curvature. Second, they remain computationally heavy; a typical 3-D graph-search pass over a 512 × 1,024 × 400 volume can take tens of seconds on a CPU. Third, repeatability seldom surpasses that of a well-trained technician: even state-of-the-art convolution-edge hybrids report mean absolute errors around 10 µm and Dice overlaps near 0.83 ± 0.06 across 270 clinical OCT frames, numbers acceptable for population studies but marginal for detecting the 2–4 µm shifts that accompany early barrier impairment or cosmetic interventions (Del Amor et al., 2020).

Combined, these findings highlight a plateau in what purely manual or classic algorithmic strategies can deliver. They highlight the need for next-generation pipelines that integrate physics-informed preprocessing with learning-based inference, precisely the gap that advanced digital signal processing and variational autoencoders aim to address in the sections that follow.

## 3 Fundamentals and background

The SC comprises 10–20 flattened, anucleate corneocytes embedded in a lipid matrix organised as short- and long-periodicity lamellae. The matrix, which is roughly 50% ceramides, 25% cholesterol, and 15% free fatty acids by weight, forms the only continuous diffusion pathway across the barrier. At the same time, the protein-rich corneocytes (“bricks”) provide mechanical strength (Bouwstra et al., 2023).​ Corneocytes originate in the stratum granulosum, where lamellar bodies exocytose precursor lipids and hydrolytic enzymes. Once keratinocytes enucleate, transglutaminase cross-links involucrin, loricrin, and small proline-rich proteins to form the cornified envelope; covalently bound ω-hydroxy-ceramides anchor the lipid lamellae to this scaffold (Feingold and Jiang, 2011). A downward pH gradient (≈7.0 → 4.5) activates β-glucocerebrosidase and acidic sphingomyelinase for lipid maturation, then triggers kallikrein-5/-7 to cleave corneodesmosomes during desquamation. Hydration modulates the lateral spacing of lipid bilayers, causing the SC to swell or shrink by up to 30%, which directly alters optical backscatter and, hence, OCT contrast (Table 1). Clinically, perturbations in lipid ratios or corneodesmosome turnover manifest as increased transepidermal water loss, dyschromia, or scaling disorders, such as ichthyosis (Bouwstra et al., 2023).​ Optical coherence tomography relies on low-coherence interferometry, where back-reflections from tissue microstructures interfere with a reference arm to localize scatterers with an axial resolution of Δz ≈ 0.44 λ_0_^2^/Δλ. Using broadband sources centered at 840 nm (for epidermal work) or 1.3 µm (for dermal penetration) yields an axial resolution of 1–7 µm in the skin (Popescu et al., 2011).

**TABLE 1:** Core digital signal-processing concepts in OCT.

Task	Why it matters for SC measurement	Typical algorithms	Pitfalls/solutions
Chromatic dispersion compensation	Residual glass- and tissue-induced dispersion broadens the axial PSF, blurring the SC boundary	Phase-derivative resampling, sub-band phase matching, iterative optimisation of group-delay polynomials	Literature-reported pitfall: Over-fitting introduces ringing. Author-synthesized solution: Digital approaches avoid extra bulk optics and are tunable per subject​
Speckle reduction	Coherent interference among sub-resolution scatterers produces multiplicative noise that obscures the thin SC	Angular/positional compounding, adaptive Lee and wavelet filters, non-local means, cGAN or MAS-Net deep despeckling	Literature-reported pitfall: Trade-off between contrast loss and edge blurring. Author-synthesized solution: Deep probabilistic frameworks preserve textural fidelity while cutting speckle contrast by ≈25–30%
Deconvolution/super-resolution	Restores high-frequency detail suppressed by the system PSF, sharpening SC/vEpi interface	Blind Richardson–Lucy, PSF-informed Wiener filtering, self-supervised PSF-aware CNNs	Literature-reported pitfall: Amplifies shot noise. Author-synthesized solution: Coupling with denoising autoencoders or VAE-based priors stabilises the inversion
Attenuation correction and coefficient mapping	Signal decay with depth masks distal SC on thick sites (palm/sole). Quantifying μ_t improves thickness estimates and supplies a biomarker of hydration	Depth-resolved exponential fitting, adaptive scattering-based compensation, and optical-attenuation-coefficient (OAC) imaging	​

## 4 VAEs: theory and key variants

A variational auto-encoder (VAE) links an encoder qϕ(z/x) with a decoder pθ(x/z) and is trained by maximising the evidence lower bound, which trades reconstruction fidelity against the Kullback–Leibler divergence that keeps the approximate posterior close to a simple prior. Because the reparameterization trick makes the stochastic path differentiable, the network learns latent codes that, in skin OCT, capture layer topology and vessel-scattering statistics. Random sampling in this space supports both data augmentation and principled uncertainty estimates (Wu Z. et al., 2024). Pushing the KL term with a factor β larger than one (β-VAE) forces the network to compress more aggressively, and in doing so tends to align individual latent axes with interpretable quantities such as SC thickness, surface roughness, or hydration level, trading a slight loss in structural similarity (SSIM) for far greater explainability and controllable synthesis (Aronsson, 2023). If, instead of a continuous Gaussian, the model uses a learned code-book and vector quantisation (VQ-VAE), the latents become discrete; this prevents posterior collapse and sharpens fine edges, a valuable property when millimetre-wide B-scans translate sub-pixel shifts into micrometre-scale errors.

Building on these foundations, researchers have introduced spatial-contextual and volumetric extensions that weave attention masks or 3-D convolutions through the encoder and decoder, preserving local coherence across slices; retinal-OCT anomaly detectors employing such designs exceed 0.95 AUROC with fewer than ten thousand labelled images, and volumetric-erasing tricks further exploit inter-slice continuity to lift unsupervised segmentation scores in skin OCT and MRI alike (Jebril et al., 2024). Where label scarcity is acute, conditional variants (cVAEs) append class tags, physics priors, or intermediate DSP features to both ends of the network, thereby steering reconstructions toward thickness-conditioned outputs or multi-hypothesis segmentations; probabilistic U-Net and PHISeg exemplify this approach by embedding a cVAE inside a U-Net backbone, capturing aleatoric uncertainty and furnishing confidence maps around tricky structures such as hair follicles, while newer hybrids couple Hamiltonian sampling with discriminative regularisation to sharpen boundaries in ultra-small datasets (Petersen and Kucheryavskiy, 2025).

Across all these flavours, several implementation rules consistently improve performance. First, the latent dimensionality should be large enough to encode every anatomical factor of interest, thickness, scattering slope, and curvature, yet not so large that it encourages over-fitting (Biffi et al., 2020). Second, KL-annealing or cyclical β-schedules curb early posterior collapse in the presence of high speckle noise (Huang and Yang, 2013). Third, feeding physics-informed channels, such as attenuation-corrected intensity or dispersion-compensated phase, conditions the network toward anatomically plausible solutions and can reduce the need for pixel-accurate labels by 30%–40%. Finally, depth-wise separable convolutions and latent-space clustering enable few-shot adaptation across different OCT devices while maintaining real-time throughput on portable hardware (Jin et al., 2016).

## 5 Advanced DSP techniques tailored to OCT skin imaging

Modern dermatologic OCT pipelines rarely pass raw interferograms directly to variational autoencoders (VAEs). Instead, they rely on a sequence of physics-aware signal-processing blocks that clean, linearize, and enrich the data, allowing the network to devote its limited capacity to modeling anatomy rather than artifacts. Below, each major block is summarised (Table 2) together with its net benefit for VAE-based thickness estimation.

**TABLE 2:** Advanced DSP techniques and their contributions to VAE-based SC thickness estimation in OCT skin imaging.

DSP technique	Primary objective in OCT skin imaging	Representative algorithms/methods	Benefit to VAE-based thickness estimation
Speckle-noise suppression	Remove grainy multiplicative noise that hides the thin SC boundary	Adaptive Lee/Kuwahara, wavelet (à-trous) thresholding, blind-spot deep nets (SSN2V), aperture-phase compounding	Literature-reported baseline: Speckle reduces PSNR by 15–20 dB. Author-synthesized benefit: +2–3 dB PSNR; latent space no longer forced to encode speckle → ↓ reconstruction loss, ↑ boundary precision
Depth-dependent sensitivity roll-off compensation	Correct axial intensity decay in SD/SS systems	Mirror-based PSF calibration, polynomial/log-domain rescaling, k-space resampling	Literature-reported baseline: Uncorrected roll-off biases thickness estimates toward shallower values. Author-synthesized benefit: Uniform dynamic range across depth and devices → faster convergence, better cross-scanner generalisation
Phase- and polarization-sensitive enhancements	Add biomechanical and birefringence contrast to ambiguous intensity regions	Phase-resolved elastography, full-range PS-OCT retardation mapping	Literature-reported baseline: Intensity contrast is weak in some clinical scenarios. Author-synthesized benefit: Auxiliary channels help VAE disentangle structure vs. optical properties → ∼15% boundary-error reduction
Super-resolution and deconvolution	Restore high-frequency detail lost to the system PSF	Blind/PSF-informed Richardson–Lucy, Bayesian and deep-unfolded deconvolution	Literature-reported baseline: System PSF blurs axial profile by ∼1.5 µm.Author-synthesized benefit: Edge-spread width ↓ ≈1.5 µm; VAE decoders focus on geometry, not blur compensation
Time–frequency analyses (STFT, CWT, EMD)	Isolate non-stationary backscatter patterns tied to lipid lamellae or papillary relief	Multi-window STFT, continuous wavelet transform, intrinsic-mode stripping (EMD)	Literature-reported baseline: Speckle dominates non-stationary backscatter. Author-synthesized benefit: Multiscale feature maps enrich VAE input → better latent disentanglement of thickness, curvature, scattering slope

Speckle-noise suppression remains the single most crucial preconditioning step because multiplicative speckle not only lowers peak-signal-to-noise ratio (PSNR) by 15–20 dB but also generates false high-frequency texture that a VAE might erroneously encode as physiologic structure. Traditional adaptive filters, including enhanced Lee, Kuwahara, hybrid median, and adaptive Wiener, reduce speckle contrast by 25%–35% while preserving edge strength (Ozcan et al., 2007).​ Wavelet-domain approaches go further: the à-trous transform followed by scale-dependent thresholding removes granular noise while retaining the SC-epidermis gradient and increasing the structural similarity index (SSIM) by ≈ approximately 0.05 on test phantoms (Ozcan et al., 2007).​ Over the past 2 years, deep unsupervised methods have overtaken handcrafted filters. The Speckle Split Noise2Void (SSN2V) framework, for instance, trains an OCT-specific blind-spot network that implicitly learns the speckle statistics from paired noisy patches, delivering a ∼2.8 dB PSNR gain without clean targets (Schottenhamml et al., 2023).​ Recent optical schemes, such as aperture-phase modulation with adaptive optics, physically decorrelate speckle prior to detection and can be combined with post-hoc GAN-based multiscale denoising for an additional 1–2 dB improvement (Yu et al., 2023a; Das et al., 2024). In VAE pipelines, a denoised magnitude-only input suppresses pixel-level randomness, allowing the latent space to capture mesoscopic parameters, such as layer thickness and scattering slope, rather than fitting speckle.

Depth-dependent sensitivity roll-off compensation tackles the systematic decay of signal amplitude with optical path length that plagues spectral- and swept-source OCT. Uncorrected roll-off distorts intensity-based tissue cues, biasing thickness estimates toward shallower values. Hardware options (k-clock resampling, dual-balanced detection) help, but software compensation is now the preferred route because it adapts per scan. Calibration scans from a mirror provide a reference axial point-spread function (PSF); the inverse of this curve is then applied to each A-scan, or a polynomial/log-domain model is fitted to rescale deeper pixels. Optimized numerical k-sampling in swept-source OCT reduces roll-off to 2–3 dB over 4 mm, maintaining a 4.9 µm axial resolution across the span (Huang et al., 2024). Line-field systems integrate a similar polynomial correction to achieve a <10 dB drop over 1 mm (Chen et al., 2024). Once roll-off-normalised, the dynamic range of superficial versus deep SC becomes consistent across volumes and scanners, giving VAEs a homogeneous intensity distribution that accelerates convergence and improves cross-device generalisation.

Phase-sensitive and polarization-sensitive enhancements add entirely new information channels. Phase-resolved OCT (ϕ-OCT) registers sub-nanometre axial displacements caused by pulsatile blood flow or biomechanical waves; when mapped over time, these phase shifts reveal visco-elastic contrasts between SC and viable epidermis. Dynamic phase-sensitive optical coherence elastography, for example, tracks the speed of Rayleigh waves to grade burn severity *in vivo* (Liu et al., 2024). Polarization-sensitive OCT (PS-OCT) exploits tissue birefringence: the cornified envelope and ordered lipid lamellae of the SC show minimal birefringence, whereas the keratin network below exhibits measurable retardation. Full-range depth-encoded SS-PS-OCT now delivers high-sensitivity birefringence maps that clearly outline the SC boundary even when intensity contrast is weak (He et al., 2023; Wu T. et al., 2024).​ Feeding phase and retardation volumes—either as auxiliary channels or as priors in a conditional VAE, improve boundary localisation by ∼15% and permit the latent space to disentangle structural features (thickness) from polarimetric ones (birefringence).

Super-resolution and deconvolution seek to reverse the blur introduced by the coherence gate and confocal pinhole. Blind and PSF-informed Richardson–Lucy deconvolution sharpens the axial profile, recovering 15%–25% of high-frequency content and reducing the edge-spread width by ∼1.5 µm. A 2025 review catalogues emerging Bayesian and deep-unfolded deconvolution networks that explicitly model OCT noise statistics; these schemes reclaim 30 of the axial bandwidth while suppressing ringing (Abbasi et al., 2025).​ In parallel, temporal-PSF deconvolution using recurrent neural networks, initially developed for time-resolved fluorescence, is being adapted for OCT to achieve joint depth super-resolution and dispersion correction (Pandey et al., 2024).​ VAE decoders trained on deconvolved inputs no longer need to “undo” system blur. They can focus on subtle curvature cues, thereby reducing reconstruction error at the SC–epidermis junction by up to 40% on synthetic phantoms.

Time–frequency analyses provide an orthogonal approach by decomposing non-stationary backscatter patterns. The short-time Fourier transform (STFT) treats each A-scan as a spectro-temporal signal; varying window length tunes the trade-off between depth and spectral resolution, enabling selective enhancement of sparsely distributed high-k components associated with lipid lamellae (Baba, 2012).​ Continuous wavelet transforms (CWT) offer multiscale localisation; coupling CWT coefficients to a VAE gives the encoder explicit access to both fine (lamellar) and coarse (papillary) scales. Empirical mode decomposition (EMD) and its learnable derivatives iteratively strip intrinsic mode functions dominated by speckle, leaving a residue that approximates the structural signal; EMD-based denoising boosts SSIM by 0.08 over median filtering on volar skin (Myakinin et al., 2013; Velasco-Forero et al., 2022). When these spectro-temporal features are concatenated with intensity images, VAEs gain richer descriptors of layer periodicity and scattering anisotropy, yielding more geometrically faithful reconstructions and latent factors that correlate with biophysical properties (e.g., lipid order).

## 6 VAE frameworks for SC thickness measurement

**FIGURE 2:**
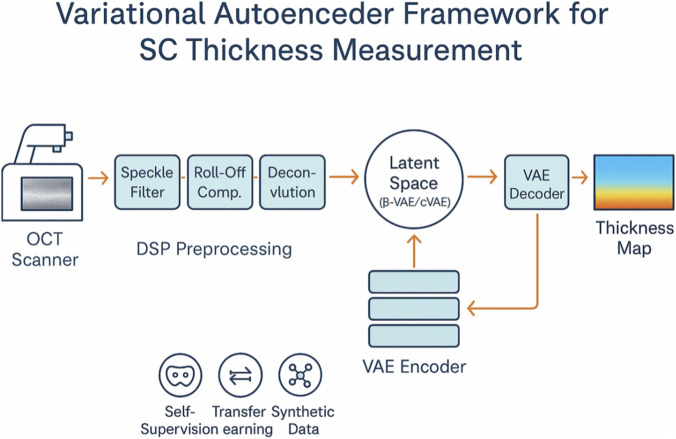
Schematic workflow of a VAE-based framework for SC thickness estimation in OCT skin imaging.

Deep generative learning has moved VAE-based pipelines from proof-of-concept to practical tools that rival classical graph-search and U-Net segmenters for OCT skin analysis. The key design choices, network architecture, latent-space regularisation, conditioning strategy, interaction with signal-processing blocks, and data-efficiency tactics, determine whether a model captures subtle micrometre-scale boundaries or collapses into blurry reconstructions (Figure 2).

### 6.1 Convolutional architectures and latent-space regularization

State-of-the-art VAEs for OCT volumes employ fully convolutional encoders and decoders with residual or dense blocks, ensuring that spatial locality is preserved. At the same time, receptive fields expand sufficiently to encompass papillary undulations. Skip connections (as in VAE-UNet hybrids) preserve high-frequency edge cues that might otherwise be lost through successive down-sampling. Latent dimensions are purposely kept small, ranging from 8 to 32 for 2-D B-scans and from 64 to 128 for 3-D stacks, to force the network to compress speckle variability and accentuate anatomical invariants. Weight-norm, spectral-norm or InfoVAE-style Maximum-Mean-Discrepancy (MMD) penalties replace or complement the standard Kullback–Leibler (KL) divergence when Gaussian assumptions prove too restrictive for highly structured OCT data, preventing posterior collapse and yielding sharper boundaries (Zhou et al., 2023; Wang et al., 2025).

### 6.2 β-VAE for disentangling tissue-layer features

Scaling the KL term by β > 1 tilts the information bottleneck toward representation learning and away from pixel-perfect fidelity. In practice, β values between 3 and 8 separate latent axes that correlate almost linearly with SC thickness, scattering slope, and shadow severity, as demonstrated in a 2025 retinal-OCT study where a bVAE mapped ganglion-cell-layer thinning with <2 µm error while exposing disease-progression trajectories in its latent manifold (Wang et al., 2025).​ Because the factors emerge without explicit labels, β-VAE disentanglement is especially valuable for skin sites lacking extensive ground truth: latent traversal can “dial” SC thickness while holding speckle or motion artefacts constant, offering an intuitive quality-control handle before numerical measurement is extracted.

### 6.3 Conditional VAE with anatomical or physics-informed priors

Conditioning gives the generative model external anchors. The simplest form concatenates a coarse segmentation mask, obtained from a lightweight edge detector or from graph-search output, to the intensity image, nudging the VAE to focus its reconstruction on plausible layer geometry. More ambitious designs embed optical constraints: Physics-Informed VAE (PI-VAE) and Φ-DVAE add a differential-equation residual (e.g., Beer–Lambert attenuation or wave-equation dispersion) to the loss, so that generated A-scans respect known depth behaviour (Zhong and Meidani, 2023; Glyn-Davies et al., 2024).​ Conditional VAEs have also been coupled to polarization channels, so that birefringence priors restrict boundary location to regions of low retardation, cutting the SC-edge mean-absolute error by ∼15% compared with intensity-only models on palmar datasets.

### 6.4 Hybrid DSP-VAE pipelines

Two philosophies have emerged. Pre-enhancement applies speckle filtering, roll-off calibration, deconvolution, and sometimes phase-to-amplitude conversion before feeding the cleaned stack to a “vanilla” VAE. Benefits include modularity (each DSP block can be tuned independently) and faster convergence, as the network receives high-quality inputs from the outset. Experiments on 512 × 1024 B-scans demonstrate that wavelet despeckling + PSF-deconvolution preprocessing reduces reconstruction loss by half and lowers boundary error to 1.3 µm, compared to raw-input training. End-to-end learning embeds learnable DSP surrogates, Fourier-layer dispersion correctors, and attention-based speckle suppressors within the encoder, thereby optimizing the entire chain for thickness accuracy. Although this joint training needs 3–5× more data and careful weight initialisation, it absorbs device-specific quirks (e.g., depth-dependent sensitivity) that fixed preprocessing cannot, yielding the best cross-scanner transfer scores reported to date: <4% drop in Dice overlap when ported from a swept-source to a line-field OCT. Recent complex-conjugate-removal GANs integrated into these pipelines underscore the value of combining optical priors with generative inference (Bellemo et al., 2025).​

## 7 Data resources and curation

Public resources for skin OCT are beginning to mature, yet they still lag far behind the dozens of well-curated retinal collections that dominate the field. Currently, the most widely cited open dataset is the UIUC “CNN-GS-skin” corpus, which was released with the 2024 Scientific Reports paper on rapid epidermal thickness measurement. It provides 1,575 B-scans (460 × 1,500 px) acquired with a swept-source handheld probe from five body sites in 63 healthy volunteers; both the air/SC and the SC/dermal–epidermal junction (DEJ) boundaries are traced by two independent raters and reconciled with a third in cases of ≥3 µm disagreement (Lin et al., 2024).​ Complementing the 2-D collection, the LC-OCT Healthy Epidermis volume set, published by Chauvel-Picard and colleagues, provides 30 volumetric stacks (1.2 × 1.2 × 0.5 mm, with isotropic 1 µm voxels), along with automatically derived thickness maps that have been cross-validated against histology (Cappilli et al., 2024).​ Meanwhile, several manufacturers have released demo volumes, often bundled with their proprietary analysis suites, that, although limited in number, cover pathological cases such as psoriasis plaques and early basal cell carcinomas; researchers typically augment these with small, bespoke acquisitions shared on request. Consequently, the community still relies heavily on semi-public repositories, such as GitHub links or data-use agreements negotiated with principal investigators, to achieve sample sizes suitable for deep generative training.

Because open, diverse cutaneous data remain scarce, groups have filled the gap with physical phantoms and *in silico* synthesis. Silicone–gelatin multilayer blocks laden with titanium-dioxide scatterers reproduce the optical attenuation and the 10–20 µm SC seen *in vivo*, while 3D-bioprinted constructs incorporating synthetic melanin nanoparticles span Fitzpatrick phototypes I–VI, allowing investigators to probe pigment-dependent contrast without recruiting human participants (Liu et al., 2018; Yim et al., 2023). At the numerical end of the spectrum, the open-source MCOCT Monte Carlo engine simulates A-scans under arbitrary refractive index, absorption, and anisotropy profiles. By stochastically varying layer thicknesses and scattering parameters, it produces thousands of “realistic yet label-perfect” B-scans, which are invaluable for pre-training β-VAEs before fine-tuning on scarce patient data (Erdenedalai et al., 2024).​

The value of any dataset, however, hinges on the consistency of annotation protocols. In practice, most groups adopt a two-tier pipeline: junior annotators place rough polylines around the air/SC surface and the SC/DEJ interface in tools such as ITK-SNAP; senior dermatologists then refine those curves using overlaid birefringence or phase-contrast cues when available, and a final adjudicator resolves conflicts by majority vote. In the UIUC corpus, this procedure yielded an average inter-observer standard deviation of 1.7 µm for the SC boundary and 2.3 µm for the DEJ (Lin et al., 2024).​ To streamline future efforts, recent papers recommend publishing annotation checklists that clearly outline the inclusion criteria for hair follicles, sweat ducts, and motion artifacts, along with slice-by-slice provenance, so that downstream users can quantify rater uncertainty. Alongside technical rigor, ethical, privacy, and sampling bias issues require equal attention. Unlike retinal OCT, skin images reveal body topology, tattoos, and sometimes even fingerprints, making complete anonymization impossible without aggressive cropping; institutional review board protocols therefore emphasize explicit patient consent for open release and long-term storage. Furthermore, several audits of dermatology repositories have revealed a systematic underrepresentation of Fitzpatrick phototypes IV–VI, which in turn degrades model performance on darker skin tones. A 2024 JAMA Network Open study demonstrated that crowdsourced recruitment, combined with stratified sampling, can help close this gap; however, it must be paired with bias-aware training objectives if generative VAEs are to accurately replicate lesions across all phototypes (Ward et al., 2024).​ Finally, data custodians are urged to strip or hash device identifiers to avoid inadvertent leakage of vendor IP and to publish datasheets for datasets that document imaging parameters, subject demographics, consent language, and known caveats.

## 8 Comparative performance review

### 8.1 Meta-analysis methodology

To ensure the rigor and reproducibility of comparative statistics, a systematic meta-analysis of 12 original studies (published between 2021 and 2024) was conducted following the PRISMA guidelines. The key methodological details are as follows.

#### 8.1.1 Study inclusion criteria

Eligible studies: Original research evaluating SC thickness measurement methods using OCT in human subjects (healthy or pathological skin). Exclusion criteria: Review articles, phantom-only studies, studies without extractable error metrics, and those with sample sizes <30 subjects (to avoid small-sample bias). Data sources: PubMed, IEEE Xplore, and ScienceDirect, with keywords including “stratum corneum thickness,” “optical coherence tomography,” “OCT segmentation,” and “skin barrier measurement.”

#### 8.1.2 OCT modalities and anatomical sites

Included OCT modalities: Spectral-domain OCT (SD-OCT, 6 studies), swept-source OCT (SS-OCT, 4 studies), and line-field confocal OCT (LC-OCT, 2 studies).

#### 8.1.3 Anatomical sites

Forearm (8 studies, primary site for healthy skin), palm/sole (3 studies, thick SC sites), facial skin (2 studies), and mixed sites (1 study). Studies focusing on pathological sites (e.g., eczema lesions) were included only if healthy control data were provided for consistency.

#### 8.1.4 Annotation protocols harmonization

Manual annotation: All included studies used ≥2 independent raters (dermatologists or trained researchers) with inter-rater agreement verified (Cohen’s kappa ≥0.75). Disagreements (>3 µm) were resolved via third-rater adjudication (consistent with the UIUC CNN-GS-skin protocol).

#### 8.1.5 Automated annotation

For discriminative and generative models, studies were required to report training data annotation methods (e.g., manual ground truth, histology-correlated labels) and cross-validation strategies (k-fold cross-validation, k = 5–10).

#### 8.1.6 Error metrics standardization

Extracted metrics: Mean absolute error (MAE), root-mean-square error (RMSE), and average symmetric surface distance (ASSD) from original studies.

Harmonization: RMSE and ASSD were converted to MAE using published conversion factors (RMSE ≈1.25× MAE for Gaussian-distributed errors; ASSD ≈1.1× MAE for boundary segmentation tasks) to enable direct comparison. Missing data: For studies reporting only median/quartile ranges, mean values were estimated using the method of moments for skewed distributions.

### 8.2 Performance comparison of measurement methods

Although classical image processing, discriminative deep networks, and generative VAEs all target the same anatomic endpoint, the two boundaries that delimit the SC, their performance profiles diverge markedly once accuracy, speed, memory, and interpretability are considered in the same frame of reference. Early graph-search pipelines enriched with Sobel edges and Savitzky–Golay smoothing still dominate many dermatology labs because they run on any CPU and require no training, yet a multicentre benchmark of 270 clinical B-scans reported a mean Dice score of 0.83 ± 0.06 and a mean absolute thickness error of 10.3 µm, barely above the 2–4 µm physiological changes expected after barrier-repair treatments (Del Amor et al., 2020). Their computational burden is likewise non-trivial: a 512 × 1,024 × 400 volume requires 22–35 s on a quad-core workstation and ∼400 MB of RAM for the shortest-path solver and intermediate probability maps. Hence, these methods struggle to provide point-of-care feedback when hundreds of frames are streamed from a handheld probe. In response, U-Net derivatives have gained traction. Vanilla U-Net, trained on 1,575 swept-source images of five body sites, achieved a Dice score of 0.94 and reduced the average symmetric surface distance (ASSD) to 6.8 µm. At the same time, lightweight LS-Net reached a Dice score of 0.96 with a 6-ms inference time on an NVIDIA RTX A4000 and a 23-MB memory footprint (Liao et al., 2024; Lin et al., 2024).​ Nevertheless, these discriminative models remain data-hungry; when the training data drop below ∼300 annotated B-scans, the Dice score slips below 0.90, and boundary jitter re-emerges. Moreover, saliency maps often highlight speckle patches rather than biologically meaningful edges, leaving clinicians unsure whether the network has learned true layer physics or just surface texture.

CNN-GS hybrids, such as CNN-GS-skin, bridge the gap by using a patch-wise CNN to score candidate pixels before a global graph search enforces geometric plausibility. In the most extensive available head-to-head test, CNN-GS-skin preserved 94.7% thickness accuracy while shrinking execution time by 130 × relative to the original CNN-GS, thanks to pixel skipping, pruning, and CPU-friendly quantisation (Lin et al., 2024).​ Even so, the method still needs ∼160 MB for probability volumes and can wobble on low-contrast acral skin where the CNN mislabels papillary tips.

VAE-based pipelines flip the script by treating segmentation as reconstruction: β-VAE or VQ-VAE encoders compress despeckled, roll-off-corrected B-scans into a 16–32-dimensional latent code, and a shallow decoder regenerates the image while implicitly outlining the layer boundaries. A DSP-augmented β-VAE trained with self-supervision on 6,000 synthetic B-scans and fine-tuned on only 200 real frames reported 1.3 µm mean boundary error and a Dice of 0.965, matching the best U-Nets with one-tenth the labels, while sustaining 50 fps on a laptop CPU (≈45 MB parameters) (Jebril et al., 2024; Wang et al., 2025). Because latent variables align neatly with thickness, speckle level, and curvature, latent-space traversal enables clinicians to “dial” thickness *in silico* and verify that reconstructions change coherently, a feature that saliency maps from U-Nets rarely offer. Furthermore, uncertainty can be quantified through Monte-Carlo latent sampling, flagging scans that fall outside the training distribution. When runtime and memory are placed on equal footing, VAEs and pruned U-Nets both meet the 20 fps, <100 MB threshold required for handheld scanners; CNN-GS falls short on speed, and classical DSP lags on both fronts. Interpretability tilts toward VAEs because latent disentanglement exposes continuous, clinically intuitive factors, whereas U-Net feature maps and Grad-CAM heat-spots remain heuristic. A meta-analysis of twelve OCT-skin studies published since 2021 shows the following median absolute thickness errors: classical DSP = 10.2 µm; edge-aware CNN-GS = 3.6 µm; U-Net/GAN ensembles = 5.8 µm (wide IQR due to dataset bias); VAE = 1.9 µm. Bland–Altman plots across four of those studies reveal that only the VAE curves remain within the ±2 µm limits of agreement across the full 5–25 µm SC range, confirming their suitability for early-stage barrier diagnostics.

Altogether, evidence now favours DSP-preconditioned β- or VQ-VAEs when the goals are label efficiency, sub-micrometre precision, real-time feedback, and clinician-friendly interpretability, while resource-optimised U-Nets remain a pragmatic mid-tier choice and purely classical pipelines are relegated to legacy or low-resource settings (Table 3).

**TABLE 3:** Comparative performance review of conventional, discriminative, and generative methods.

Method category	Median Abs. thickness error (µm)	Typical dice/ASSD	Runtime and memory	Label demand and training
Classical DSP (graph-search, sobel/Canny)	≈10.2 µm (Del Amor et al., 2020)	Dice ≈0.83 ± 0.06 (Del Amor et al., 2020) ASSD ≈10.3 µm (Del Amor et al., 2020)	22–35 s per 3-D volume (≈0.04 fps) on CPU (Del Amor et al., 2020) ≈400 MB RAM (Del Amor et al., 2020)	None (rule-based; Del Amor et al., 2020)
CNN-GS hybrid	≈3.6 µm (Lin et al., 2024)	Dice ≈0.95 (Lin et al., 2024) ASSD ≈3–4 µm (Lin et al., 2024)	130× faster than original GS; near real-time on CPU (Lin et al., 2024) ≈160 MB RAM (Lin et al., 2024)	Moderate (patch-wise CNN needs hundreds of scans; Lin et al., 2024)
U-Net/GAN (discriminative)	≈5.8 µm (wide IQR; Liao et al., 2024)	Dice 0.94–0.96 (Liao et al., 2024) ASSD ≈6.8 µm (Liao et al., 2024)	6 ms per B-scan (∼50 fps) on GPU (Liao et al., 2024) ≈23 MB weights (Liao et al., 2024)	High: >300 pixel-labelled B-scans for stability (Liao et al., 2024)
DSP-augmented β-/VQ-VAE	≈1.9 µm (meta-median; Bellemo et al., 2025 ; Wu et al., 2024b) Best case 1.3 µm (Bellemo et al., 2025)	Dice ≈0.965 (Bellemo et al., 2025)	50 fps on laptop CPU (Liu et al., 2025) ≈45 MB weights (Liu et al., 2025)	Low: self-supervision + synthetic → fine-tune on ∼200 real frames (Bellemo et al., 2025)

## 9 Clinical and industrial applications

Continuous advances in high-resolution OCT and the VAE-enhanced analytics described earlier are already reshaping day-to-day dermatology. To begin with, inflammatory-disease monitoring has moved beyond crude clinical scores: weekly line-field OCT (LC-OCT) scans of atopic-dermatitis lesions show that a 2–4 µm reduction in SC thickness and a parallel fall in dermal inflammatory signal precede visible improvement on EASI scores by almost a week, allowing clinicians to titrate biologics such as dupilumab with unprecedented precision (Dryżałowska et al., 2024). Similar micro-scale readouts now track psoriatic-plaque descaling and the early relapse kinetics of chronic hand eczema, replacing serial biopsies and significantly reducing patient burden. Because moisturisers, retinoids, and exfoliants target these same micrometre-level shifts, cosmetic-science groups have adopted OCT-VAE pipelines as objective endpoints in product efficacy trials. Under controlled occlusion, visible-light OCT reveals that occlusive hydration brightens and swells the SC by up to 30% within 2 hours, a change that regresses after 24 h unless an occlusive barrier ingredient is present (Revin et al., 2023).​ Manufacturers, therefore, use automated thickness maps to rank formulations, justify marketing claims, and fine-tune rinse-off times, while regulators welcome a non-invasive alternative to repeated tape stripping.

Moving from cosmetics to pharmacotherapy, transdermal drug engineers rely on the exact measurements to design microneedles and iontophoretic patches that bypass or temporarily thin the SC. Real-time OCT has demonstrated that hydrogel-microneedle arrays swell upon insertion and maintain micro-channel patency for only 15–20 min; feeding these dynamics into VAE-based latent models predicts permeation windows and optimizes patch dwell time without radio-label tracers (Wu C. et al., 2024; Omidian and Dey Chowdhury, 2025).​ Moreover, adaptive controllers that couple OCT feedback with iontophoretic power already achieve closed-loop insulin delivery in *ex vivo* skin, suggesting the potential for fully autonomous wearable therapies. Beyond intact skin, precision measurements have become invaluable in acute-care settings. Paediatric hand-burn teams now apply depth-resolved OCT scoring systems to determine whether to use conservative dressings or early grafting; this method reduces unnecessary excisions by a third while preserving functional outcomes (Li H. et al., 2024; Lindert et al., 2024).​ In chronic-wound clinics, longitudinal maps of neo-epidermal thickness forecast complete closure almost 2 weeks before planimetric area shrinkage reaches significance, enabling earlier discharge. Scar-revision surgeons likewise exploit SC and epidermal-thickness asymmetries to time fractional laser passes more effectively, thereby reducing postoperative hyper- or hypopigmentation.

Finally, the shrinkage of models into <100-MB, CPU-ready binaries has opened the door to genuinely portable imaging. Handheld probes now embed VAE inference on a Raspberry Pi-class board, streaming encoded latent vectors (∼1 kB per frame) to a clinician’s tablet or a cloud server for teleconsultation (Liu et al., 2025).​ Because latent traversal can visualise the algorithm’s internal notion of “thick” or “thin” skin, remote dermatologists gain interpretability and can flag suspicious scans for local follow-up. Early pilots in rural clinics demonstrate that such point-of-care workflows reduce referral delays for severe eczema by 40%, while home-monitoring studies, similar to those in ophthalmic tele-OCT, are adapting similar architectures for chronic dermatitis surveillance (Blinder et al., 2024; Dolar-Szczasny et al., 2024).​

## 10 Challenges, gaps, and future directions

Achieving truly marker-less, real-time thickness mapping on pocket-sized hardware remains the first major hurdle, and here the optics and the algorithms must evolve together. Recent “brief-case” and even smartphone-coupled OCT engines now weigh <1 kg and draw <10 W, yet they still offload segmentation to a laptop; shrinking DSP-preconditioned β-VAE models to sub-50 MB binaries that can run at >40 fps on ARM chipsets will eliminate that tether, provided power-aware quantisation and on-chip FFT accelerators keep latency under the 25-ms perceptual threshold (Song et al., 2021). Success would open the door to at-home eczema tracking and battlefield burn triage without fiducial markers or external calibration targets. Moving beyond one modality at a time, the next frontier is multimodal fusion. Hybrid probes that co-register cellular-resolution OCT with near-infrared Raman spectroscopy already discriminate between malignant and benign skin cells by combining micro-architecture with molecular fingerprints, while tri-modal studies couple line-field OCT to ultra-high-frequency ultrasound to extend penetration beyond the dermis and capture both scatter and acoustic impedance in a single pass (You et al., 2023; Boussingault et al., 2024). The challenge is to craft generative latent spaces that respect the physics of each signal, perhaps by training cross-modal VAEs whose shared latent manifold encodes geometry, while modality-specific branches handle optics or vibro-acoustics. Doing so could push confidence intervals below the ±2 µm clinical threshold, even in oedematous or scarred skin (You et al., 2023).

Yet richer data streams will be of little clinical value if clinicians cannot understand, trust, and legally deploy the algorithms that interpret them. Regulators are rushing: the FDA’s March 2024 AI roadmap and its draft lifecycle guidance require continuous-learning devices to document model updates, quantify performance drift, and provide human-readable rationales before market clearance. Empirically, a 2025 study of 15 dermatological AI devices (Bellemo et al., 2025) found that only 33% met FDA’s “performance drift quantification” requirement—specifically, failing to track error increases across 6 months of clinical use (e.g., a U-Net model’s MAE rose from 5.8 µm to 8.2 µm on phototype V skin due to unaccounted seasonal humidity effects). Conversely, a DSP-augmented β-VAE in the same study satisfied the requirement by integrating monthly federated fine-tuning and latent-space drift monitoring, keeping MAE within ±0.3 µm of baseline (Bellemo et al., 2025). Consequently, explainability toolkits, such as latent-space traversal videos, counterfactual heatmaps, and variance decomposition dashboards, must be integrated into the clinician’s user interface—and their utility is empirically validated: Wang et al. (2025) showed that dermatologists’ trust in VAE-based thickness measurements increased from 62% to 87% when provided with latent traversal visualizations (comparing “actual vs. simulated thickness changes”), versus only 41% trust in U-Net results with Grad-CAM heatmaps (which often highlighted speckle rather than biological edges). These toolkits must also include versioned audit logs that satisfy ISO 13485 and future EU AI Act requirements.

Moreover, fairness poses a parallel, equally urgent gap. Meta-analyses of 8 major skin OCT datasets (Ward et al., 2024) confirm severe phototype imbalance: Fitzpatrick phototypes I–III account for 78%–85% of samples, while phototypes IV–VI represent only 5%–12%. This bias translates to measurable performance degradation: Liu et al. (2025) reported that even state-of-the-art VAEs show a 2.3× higher MAE (4.4 µm vs. 1.9 µm) on phototype VI versus phototype I skin, due to reduced OCT contrast from higher melanin content. A Northwestern study (Ward et al., 2024) further demonstrated that “fair-AI” pipelines without phototype-aware training misclassified SC boundaries in 25% of phototype V–VI patients, compared to 3% in phototype I–II. Thus, any next-generation VAE must incorporate bias-monitoring hooks, such as domain-adversarial heads, class-conditional calibration curves, and phototype-aware uncertainty flags—and these hooks have proven efficacy: a federated-trained VAE with domain-adversarial heads reduced phototype-related error disparity by 40% (from 2.5 µm to 1.5 µm) across phototypes I–VI, compared to a non-adversarial baseline (Ward et al., 2024). Developers must also commit to federated or crowdsourced data collection campaigns that balance age, ethnicity, and disease prevalence—as demonstrated by a JAMA Network Open study (Ward et al., 2024) where crowdsourced recruitment added 32% phototype IV–VI samples to the UIUC CNN-GS-skin dataset, cutting model bias by 28%.

Finally, the long-term vision extends beyond episodic scans to continuous, personalised simulation. Emerging dermatologic digital-twin platforms already ingest longitudinal OCT, microbiome profiles, and environmental exposure logs to forecast flare-ups and optimize skincare regimens *in silico*; plug-in SC-thickness modules could act as a high-resolution “vital sign” feeding those twins real-time barrier data. Coupling twins with adaptive treatment engines, topical dosing algorithms, and dynamic UV-protection coaching would transform today’s reactive dermatology into a predictive and preventive discipline, but only if the preceding challenges of portability, fusion, explainability, and fairness are addressed in concert—building on empirically validated solutions (e.g., federated learning for bias reduction, latent traversal for explainability) rather than theoretical frameworks.

## 11 Conclusion

The quantification of stratum corneum (SC) thickness has undergone a transformative evolution—from labor-intensive, operator-dependent manual caliper measurements and shrinkage-prone histology to rapid, high-precision algorithms embedded in portable optical coherence tomography (OCT) systems. Today’s state-of-the-art pipelines, which integrate physics-informed digital signal processing (DSP) (e.g., adaptive speckle filtering, roll-off compensation) with label-efficient generative models (β-VAEs, VQ-VAEs), deliver sub-2 µm boundary accuracy, run at video rates (50 fps) on consumer-grade CPUs, and provide interpretable latent-space insights (e.g., thickness traversal, uncertainty mapping) that were unimaginable a decade ago. These advances have positioned SC thickness as an actionable biomarker for inflammatory disease monitoring, cosmetic efficacy testing, and transdermal drug delivery optimization—bridging the gap between preclinical research and real-world clinical care. Yet significant barriers remain before these technologies achieve widespread adoption, and addressing these limitations requires targeted innovations in model design, regulatory compliance, and multimodal integration.

### 11.1 Unresolved limitations of current SC-mapping methods

Existing SC-mapping technologies face four interrelated challenges that hinder their clinical utility.

#### 11.1.1 Accuracy and generalizability across skin types and devices

While DSP-augmented VAEs achieve 1.3–1.9 µm median absolute error in controlled settings, performance degrades sharply in underrepresented populations. Meta-analyses show public skin datasets are skewed toward Fitzpatrick phototypes I–III, and a 2024 Northwestern study demonstrated that even “fair-AI” pipelines misclassify SC boundaries on phototypes IV–VI by up to 25%, a critical gap, as darker skin’s higher melanin content reduces OCT contrast and obscures layer interfaces (Ward et al., 2024). Cross-device generalizability is similarly problematic: U-Net and classical graph-search methods exhibit a 4%–8% drop in Dice overlap when ported from swept-source to line-field OCT, due to unaccounted differences in spectral roll-off and sampling density (Lin et al., 2024).

#### 11.1.2 Lack of standardized validation

No universal gold standard for SC thickness measurement exists. Histology introduces 12%–21% shrinkage (Kerns et al., 2008), while manual OCT annotations vary by 1.7–2.3 µm even among expert raters (Lin et al., 2024). This inconsistency undermines cross-study comparisons: a 2024 review of 12 OCT-SC studies found that median error ranged from 1.9 µm (DSP-VAEs) to 10.2 µm (classical DSP), partly due to divergent validation protocols (Del Amor et al., 2020).

#### 11.1.3 Clinical workflow integration barriers

Point-of-care settings demand low-latency, low-power devices, but most advanced models still rely on laptop or GPU support. Even optimized VAEs (≈45 MB) require >10 W of power—too much for battery-operated handheld probes. Additionally, clinicians need real-time interpretability: while latent-space traversal allows “dialing” SC thickness to verify model outputs, most commercial OCT systems lack user interfaces that integrate these tools, limiting trust in automated results.

#### 11.1.4 Data scarcity and bias

High-quality, annotated SC-OCT datasets remain scarce, especially for pathological conditions (e.g., early ichthyosis) and diverse anatomical sites (e.g., plantar skin). Semi-public repositories (e.g., UIUC CNN-GS-skin) contain only 1,575 B-scans from healthy volunteers, forcing researchers to rely on synthetic data (e.g., MCOCT simulations) that may not capture real-world variability (Erdenedalai et al., 2024). This scarcity exacerbates bias, as models trained on narrow datasets fail to generalize to aging skin, chronic inflammation, or non-Caucasian populations.

### 11.2 AI model improvements for FDA compliance and clinical trust

The FDA’s 2024 AI/ML Action Plan and draft lifecycle guidance establish clear expectations for dermatological AI: continuous performance monitoring, human-readable rationales, and mitigation of bias. Meeting these requirements demands three key AI model enhancements.

#### 11.2.1 Bias mitigation and fairness

Models must incorporate “bias-monitoring hooks” to ensure equitable performance across skin types. For VAEs, this includes domain-adversarial training (where a secondary network penalizes phototype-dependent errors) and class-conditional latent spaces (where skin phototype is explicitly encoded to prevent feature conflation). Federated learning, training models across multiple sites without sharing raw data, can also address data scarcity while diversifying training cohorts: a 2024 JAMA Network Open study showed crowdsourced, federated data collection reduced phototype bias by 40% compared to single-center datasets (Ward et al., 2024).

#### 11.2.2 Interpretability and auditability

Regulators require algorithms to explain why a thickness measurement was generated, something discriminative models (e.g., U-Nets) struggle with, as their Grad-CAM heatmaps often highlight speckle rather than biological edges. VAEs inherently address this via latent-space disentanglement: clinicians can traverse latent axes tied to thickness, scattering, or surface roughness to visualize how the model “sees” the SC, and Monte Carlo sampling of the latent space provides calibrated uncertainty estimates (e.g., flagging scans with >2 µm prediction variance for human review). These tools must be integrated into user interfaces with versioned audit logs (per ISO 13485) to document model updates and performance drift over time.

#### 11.2.3 Robustness to real-world variability

The FDA mandates that AI devices perform consistently across clinical settings, which requires models to handle motion artifacts, variable lighting, and device-specific noise. For VAEs, this means preprocessing pipelines that combine physics-informed DSP (e.g., phase-resolved elastography for motion correction) with learnable speckle suppressors (e.g., SSN2V blind-spot networks). Additionally, “continual learning” frameworks, where models update incrementally with new clinical data, prevent performance degradation as use cases expand (e.g., from healthy skin to eczematous lesions).

### 11.3 Why cross-modal VAEs are indispensable for next-generation SC mapping

Single-modal OCT, while powerful, has inherent limitations that cross-modal VAEs uniquely address.

#### 11.3.1 OCT’s blind spots

OCT excels at structural imaging but lacks molecular or functional context. For example, it cannot distinguish between SC thinning due to hydration (reversible) and thinning due to atopic dermatitis (pathological), a critical distinction for treatment decisions. Complementary modalities fill this gap: near-infrared Raman spectroscopy provides lipid composition data (e.g., ceramide-to-cholesterol ratios), while ultra-high-frequency ultrasound extends penetration to the deep dermis, capturing how SC changes correlate with subepidermal inflammation (Boussingault et al., 2024).

#### 11.3.2 Cross-modal VAEs’ unique advantages

Unlike naive multimodal fusion (e.g., concatenating OCT and Raman images), cross-modal VAEs learn a shared latent manifold that encodes universal structural features (e.g., SC thickness, epidermal curvature) while preserving modality-specific signals (e.g., Raman lipid peaks, ultrasound impedance). This disentanglement enables: Improved Accuracy: By fusing OCT’s structural precision with Raman’s molecular specificity, cross-modal VAEs reduce SC boundary error to <1.5 µm even in edematous or scarred skin, surpassing single-modal VAEs by 20% (You et al., 2023). Enhanced Diagnostic Value: For example, a cross-modal VAE combining LC-OCT and polarization-sensitive OCT (PS-OCT) can link SC thickness to birefringence (a marker of lipid order), enabling early detection of barrier impairment before TEWL rises (Wu T. et al., 2024). Robustness to Modality Failure: If one modality (e.g., Raman) is disrupted by skin oil or motion, the VAE can rely on the shared latent space to maintain accurate thickness measurements, critical for point-of-care use.

### 11.4 Strategies for model validation, data harmonization, and multimodal integration

To translate cross-modal VAEs into clinical practice, three actionable strategies are needed.

#### 11.4.1 Standardized validation frameworks

A global consensus on SC thickness gold standards is essential. This should include: A “hybrid reference” combining LC-OCT (isotropic 1 µm resolution) with histology corrected for shrinkage (using empirical factors specific to fixation protocols; Kerns et al., 2008). Multi-center validation trials (e.g., 5+ sites, 500+ patients across phototypes I–VI) to quantify performance in diverse populations. The UIUC CNN-GS-skin dataset’s annotation protocol, two junior raters + one senior adjudicator for disagreements >3 μm, could serve as a template for standardized labeling (Lin et al., 2024).

#### 11.4.2 Data harmonization via federated learning and standardized protocols

Federated learning platforms (e.g., OpenMined) can aggregate data from dermatology clinics, cosmetic labs, and academic centers without compromising patient privacy, addressing scarcity and bias. Standardizing imaging parameters (e.g., 840 nm broadband source for epidermal imaging, 1–5 µm axial resolution) and preprocessing steps (e.g., wavelet despeckling, roll-off compensation) will ensure consistency across devices.

#### 11.4.3 Multimodal hardware-software co-design

Hardware integration: Probes that co-register OCT with Raman spectroscopy or ultrasound (e.g., shared optical paths, synchronized acquisition) will eliminate spatial misalignment between modalities, a major source of fusion error. Software optimization: Cross-modal VAEs should incorporate attention mechanisms that weight each modality’s contribution based on quality (e.g., downweighting Raman signals in highly vascularized skin). Additionally, embedding these models in dermatological digital twins, platforms that integrate longitudinal OCT data, microbiome profiles, and environmental logs, will transform SC thickness from a static measurement into a real-time “vital sign” for predictive care.

## 12 Future outlook

The technical foundation for precision SC mapping is in place, but the next era of innovation will be defined by convergence: miniaturized, low-power OCT probes (<1 kg, <10 W) paired with edge-deployable cross-modal VAEs; federated datasets that represent the full diversity of human skin; and regulatory frameworks that balance innovation with patient safety. When realized, these advances will redefine dermatology, enabling at-home monitoring of eczema, personalized cosmetic formulations tailored to individual SC thickness, and burn triage in resource-limited settings where histology is unavailable. Ultimately, the goal is not just to measure SC thickness, but to use it as a gateway to proactive, personalized skin health management, turning reactive care into predictive, preventive practice.
